# Supplementary figures and images for: Improving the Performance of a Salt Production Plant by Using Nanofiltration as a Pretreatment
Source: Membranes (Basel). 2022 Nov 25;12(12):1191. doi: 10.3390/membranes12121191 (PMC9787835; doi:10.3390/membranes12121191)

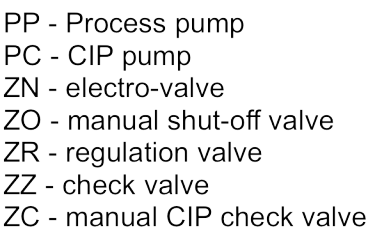

Supplement: Supplementary file 1 [file membranes-12-01191-s001.zip › Scheme S1.pdf]
